# Phospholipid and LC-PUFA metabolism in Atlantic salmon (*Salmo salar*) testes during sexual maturation

**DOI:** 10.1371/journal.pone.0233322

**Published:** 2020-05-29

**Authors:** André S. Bogevik, Edward S. Hayman, Målfrid T. Bjerke, Jens-Erik Dessen, Kjell-Arne Rørvik, J. Adam Luckenbach

**Affiliations:** 1 Division Aquaculture, Nofima AS, Fyllingsdalen, Norway; 2 Ocean Associates Inc., Under Contract to Northwest Fisheries Science Center, National Marine Fisheries Service, National Oceanic and Atmospheric Administration, Seattle, Washington, United States of America; 3 Division Aquaculture, Nofima AS, Ås, Norway; 4 Department of Animal and Aquaculture Sciences, Norwegian University of Life Sciences, Ås, Norway; 5 Environmental and Fisheries Sciences Division, Northwest Fisheries Science Center, National Marine Fisheries Service, National Oceanic and Atmospheric Administration, Seattle, Washington, United States of America; 6 Center for Reproductive Biology, Washington State University, Pullman, Washington, United States of America; University of Hyderabad, INDIA

## Abstract

The importance of dietary lipids in male reproduction are not as well understood as in females, in which dietary lipids, such as phospholipids (PL) and associated fatty acids (FA), are important structural components of the eggs and provide energy for their offspring. In mammals, lipids are suggested to be important for spermatogenesis and to structural components of the spermatozoa that could improve fertilization rates. New knowledge of how lipids affect sexual maturation in male Atlantic salmon (*Salmo salar*), an important global aquaculture species, could provide tools to delay maturation and/or improve reproductive success. Therefore, changes in testicular composition of lipids and gene transcripts associated with spermatogenesis and lipid metabolism were studied in sexually maturing male salmon compared to immature males and females. An increase in total testis content of FA and PL, and a shift to higher PL composition was observed in maturing males, concomitant with increases in mRNA levels for genes involved in spermatogenesis, FA uptake and synthesis, and production of long chain-polyunsaturated fatty acids (LC-PUFA) and PL. A particularly interesting finding was elevated testis expression of acyl-CoA synthetase 4 (*acsl4)*, and acyl-CoA thioesterase 2 (*acot2*), critical enzymes that regulate intra-mitochondrial levels of 20:4n-6 FA (arachidonic acid), which have been associated with improved cholesterol transport during steroidogenesis. This suggested that FA may have direct effects on sex steroid production in salmon. Furthermore, we observed increased testis expression of genes for endogenous synthesis of 16:0 and elongation/desaturation to 22:6n-3 (docosahexaenoic acid) in sexually maturing males relative to immature fish. Both of these FA are important structural components of the PL, phosphatidylcholine (PC), and were elevated concomitant with increases in the content of phosphatidic acid, an important precursor for PC, in maturing males compared to immature fish. Overall, this study suggests that, similar to mammals, lipids are important to spermatogenesis and serve as structural components during testicular growth and maturation in Atlantic salmon.

## Introduction

Gonads of fish undergo dramatic growth and development during sexual maturation, often increasing two orders of magnitude or more in mass [1–3]. This process is driven in part by endogenous steroid production, as well as the accumulation of lipids, primarily derived from the diet [4]. The importance of lipids and associated fatty acids (FA) in reproduction has been extensively studied in fish, particularly in females, in which they are important structural components of the eggs and an energy source for the developing embryos and larvae [4–10]. However, the importance of lipids in male reproductive development is not well understood. Better understanding metabolic factors that affect testicular maturation could provide tools to delay puberty and/or improve reproductive success in aquaculture.

Testicular lipids can come from the diet, body reserves, or may be synthesized *de novo* [11–12]. Lipids are transported through the bloodstream to target tissues as lipoproteins. Lipoprotein lipase (Lpl) is considered a key enzyme in whole-body lipid metabolism and balance, and the extrahepatic rate-limiting enzyme in the hydrolysis of circulating triacylglycerol (TAG) [13]. Lpl is also likely a key enzyme in lipid uptake by the ovary during oogenesis, as its mRNA level and enzymatic activity in European seabass (*Dicentrarchus labrax*) and shortfinned eel (*Anguilla australis*) was shown to positively correlate with ovarian lipid content and maturation stage [14, 15]. Divers et al. [15] further demonstrated that ovarian *lpl* mRNA levels were regulated by the androgen, 11-ketotestosterone, a key steroid in oocyte growth and spermatogenesis in fish [16]. Biosynthesis of androgens requires the coordinated action of several steroidogenic enzymes. Cytochrome P450 side-chain cleavage (Cyp11a), 3β-hydroxysteroid dehydrogenase/Δ^5^-Δ^4^-isomerase (Hsd3b) and cytochrome P450 17α-hydroxylase/17,20-lyase (Cyp17a) enzymes are essential for the production of precursors, while cytochrome P450 11β-hydroxylase (Cyp11b) and 11-hydroxysteroid dehydrogenase (Hsd11b) catalyze the final steps of androgen production [16–18].

The interaction between steroidogenesis and lipid metabolism goes both ways; for example, sex steroids affect lipid synthesis, and FA in turn affect steroid production [19]. This interaction stimulates the synthesis of long-chain polyunsaturated fatty acids (LC-PUFA) during sexual maturation and may broadly affect reproductive mechanisms due to high availability of these lipids. Dietary effects of FA on steroid production have been studied in several species, including European seabass [20, 21], Senegalese sole (*Solea senegalensis*) [22] and European eel (*Anguilla anguilla*) [23]. Dietary arachidonic acid (ARA; 20:4n-6) is the main precursor for the production of 2-series prostaglandins [24]. *In vitro* studies with seabass testicular cells have shown that ARA stimulates prostaglandin production in a dose- and time-dependent manner that was postulated to affect steroidogenesis [21]. In the mouse, *in vitro* studies showed that ARA is especially important in membranes of testicular Leydig cells for improved cholesterol transport [25]. Transport of cholesterol into the inner mitochondrial membrane is a rate-limiting step in steroidogenesis controlled by steroidogenic acute regulatory protein (STAR) [26]. As such, ARA has been reported to play an essential role in regulating STAR expression [27]. In addition, studies with Murine Y1 adrenocortical tumor cells have shown that the intracellular level of ARA in mitochondria is controlled by acyl-CoA synthetase long chain family member 4 (ACSL4) and acyl-CoA thioesterase 2 (ACOT2) [28]. ACSL4 sequesters free ARA to form an intracellular pool of ARA-CoA; ACOT2 then controls the release of ARA for increased STAR expression and downstream steroidogenic activity. Therefore, the dietary level of ARA is considered an important factor for reproductive development [22, 29, 30].

In addition to availability of lipids from the diet and peripheral tissues, essential FA may be hydrolyzed from testicular lipid stores. Lipase E (LIPE, also known as hormone-sensitive lipase or HSL) is an intracellular neutral lipase that hydrolyzes TAG, diacylglycerols and monoacylglycerols, as well as cholesteryl and retinyl esters from cellular stores [31]. Alternatively, FA can be synthetized *de novo* in the testis [12], catalyzed by fatty acid synthase (FASN) [32, 33]. Indeed, during the sperm maturation phase with elevated androgen production in European eel, increased testis content of 16:0 and 16:1 was observed [23], the main products of *de novo* synthesis of FA [34] and substrate for further elongation and desaturation. However, in salmonid fish, these can only be converted to monounsaturated FA [24], while LC-PUFA can only be generated from C18-PUFA via the diet or lipid stores. Essential n-6 FA (ARA) are converted from 18:2n-6, while n-3 FA (Eicosapentaenoic acid, EPA 20:5n-3 and Docosahexaenoic acid, DHA 22:6n-3) are produced from 18:3n-3. The C-18 PUFA are desaturated and elongated to C-20-22 PUFA through enzymatic reactions, with the Δ5 and Δ6 desaturase genes, *fads1* and *fads2*, respectively, introducing double bonds to the FA chain. Meanwhile, the C-18 FA are elongated to C-20 by Elovl5 and further to C-22 FA by Elovl2 [34, 35]. Still, the ability to endogenously synthesize LC-PUFA is limited in fish [5–6], and thus the main source is considered to be the diet [4].

LC-PUFA can be further esterified to lipid structures as TAG or phospholipids (PL). Phospholipids are important building blocks for cell membrane structures in the growing testis during sperm cell development [36,37], with the major PL being phosphatidylcholine (PC) and phosphatidylethanolamine (PE) in fish tissues [13]. Biosynthesis of PL has been extensively studied in intestinal tissue of Atlantic salmon (*Salmo salar*) due to the important role of PL in lipid transport [38–40]. All *de novo* production of PL proceeds from phosphatidic acid (PA) via the actions of cytidine diphosphate-choline:diacylglycerol phosphotransferases (Chpt1) and cytidine diphosphate-ethanolamine:diacylglycerol phospho-transferases (Cept1), leading to PC and PE synthesis. To our knowledge, the biosynthesis of PL during sperm maturation has not previously been studied in fishes; however, Bell et al. [41] observed at least one PUFA in almost all molecular species of PL in sperm samples collected from European seabass, Atlantic salmon, and Chinook salmon (*Oncorhynchus tshawytscha*). In a range of tissues of Japanese amberjack (*Seriola quinqueradiata*), Tanaka et al. [42] observed DHA to be the dominant FA in PC and PE, whereas ARA was highly incorporated in phosphatidylinositol (PI), which helped to reveal their functional differences in different cell types.

Although there is a clear connection between testicular lipids and endocrine factors associated with male maturation, the role of these lipids and key players involved in their metabolism are yet to be identified in fish. In this study, we sought to determine changes in mRNA levels for genes known to be important to steroidogenesis and spermatogenesis, as well as lipid metabolism, routing, uptake, and synthesis, in relation to content and composition of FA and PL in the gonads of immature and sexually maturing Atlantic salmon.

## Materials and methods

### Fish samples

Atlantic salmon used in this study were obtained from the Nofima large-scale research & development (R&D) facility collocated with Nordlaks Oppdrett AS, at the sea site, Dypingen (Kvernsundet, Bjarkøy, Troms, Northern Norway). The R&D licenses (allowance T-H-8 and T-H-19) used within this site were appointed and approved by The Norwegian Directorate of Fisheries and the farming was carried out in accordance with national guidelines, laws, and the Animal Welfare Act. Fish were treated as aquaculture production animals up to the point of tissue sampling, which was only conducted post-mortem (according to regulation FOR-2015-06-18-761). Therefore, no special approval was required from the Norwegian Food Safety Authorities for this trial, as all fish were sampled post-mortem during standard harvest by a commercial farm.

Atlantic salmon yearling smolts (S1) (AquaGen strain, AquaGen AS, Hemne, Norway) vaccinated with Pentium Forte Plus (Novartis Animal Vaccines Ltd, Essex, UK) were transferred and stocked within the R&D site on 3 May 2015 at a mean body weight of 148 g. The fish were provided feed similar to commercial diets (high-plant/low fishmeal) formulated and produced by BioMar AS (Myre, Norway) according to targeted dietary protein and lipid content defined by Nofima. The diets were produced as 3.5, 5, 7 and 10-mm pellets. The crude protein and lipid content in all pellet sizes was assessed by on-line near-infrared (NIR) analyses by BioMar and these values, in addition to the feeding periods of the different diets, are shown in Table 1. The salmon were exposed to ambient light and water temperature, with an average temperature of 8°C and minimum and maximum of 4 and 13°C, respectively, during the production phase. Since the fish were sampled from a commercial farm, most of them were harvested prior to initiation of maturation. Therefore, any maturing individuals occurred naturally and were not experimentally induced to mature.

**Table 1:** Dietary protein and lipid content used during the production phase for Atlantic salmon. Values for protein and lipid are based on the mean Near Infrared (NIR) analysis of each feed batch produced and weighted for the total amount of feed produced in each batch of the particular pellet size. The feeding periods and fish weight intervals of the different pellet sizes are also shown.

Feeding period	Pellet size used (mm)	Fish weight interval (g)	Protein (%)	Lipid (%)
May 2015—Jun 2015	3.5	75–200	46	25
Jun 2015—Aug 2015	5.0	200–500	42	29
Aug 2015—Oct 2015	7.0	500–1000	41	32
Oct 2015—Aug 2016	10.0	1000–5000	36	35

After 15–16 months (May 2015 –Aug 2016) of rearing in sea cages and achieving an average weight increase of 4.9 kg, the salmon were transported live by well-boat to the Nordlaks AS harvesting and processing plant (Stokmarknes, Norway) on the 18 and 26 August 2016. At the harvesting plant, all fish were stunned with electricity, gill arches severed, and bled out in a tank with a water temperature between -1°C and 1°C. In total, 24 fish (5.4±0.8 kg) were then randomly selected for sampling from the harvesting line after bleeding and prior to gutting: 4 immature females, 6 immature males, and 14 sexually-maturing males. Immature and maturing fish were initially identified based on the macroscopic appearance of the fish and gonads, then confirmed subsequently by gonadal histology. Length and body weight were measured, in addition to weight of the liver, viscera, and gonads for calculation of hepatosomatic index (HSI; 100 x (liver weight)/(body weight)), viscerosomatic index (VSI; 100 x (visceral weight/body weight)), gonadosomatic index (GSI; 100 x (gonad weight)/(body weight)) and gutted yield (100 x (gutted carcass weight)/(body weight)). Anterior pieces of the gonads of each fish were frozen on dry ice for chemical analysis and preserved in RNAlater (Thermo Fisher, Waltham, MA, USA) for gene expression analysis, or in 4% Formol (Q Path® - VWR, Radnor, PA, USA) for histology.

### Histology

Gonad samples in 4% Formol were dehydrated via an ethanol series, cleared with xylene substitute, and embedded in paraffin wax using an automated tissue processor (Leica TP1020 and EG1150H). Paraffin blocks were sectioned at a thickness of 5 μm using a rotary microtome (Leica RM2165), stained with hematoxylin and counterstained with eosin. Slides were viewed and photographed using a Nikon microscopy system (Eclipse 50i with DS-Fi2 camera and DS-U3 control unit) and images analyzed using NIS-Elements software. Immature and maturing fish were confirmed by gonadal histology. Females were considered immature if ovaries had not initiated vitellogenesis. Males were considered immature if only type-A spermatogonia were present (non-meiotic), and considered maturing when meiosis had progressed where spermatocytes, spermatids and/or spermatozoa were observed. Measures of the diameter of 10 randomly-selected tubular lobes of the testes per fish were performed to describe testicular growth in immature and maturing male salmon.

### Lipid analysis

Total lipids were extracted from frozen gonad samples according to Folch et al. [43]. Sub-samples of the chloroform–methanol phase were used for analysis of the FA composition of total lipids following methods described by Mason & Waller [44]. Briefly, the extract was dried under N_2_ gas and the residual lipid extract was transmethylated overnight with 2',2'-dimethoxypropane, methanolic-HCl and benzene at room temperature. Thereafter, separation and analyses of methyl esters was performed using gas chromatography (GC; Hewlett Packard (HP) 6890) with a split injector, SGE BPX70 capillary column (60 m x 0.25 mm x 0.25 μm; SGE Analytical Science, Trajan Scientific, Australia), flame ionization detector, and HP Chem Station software. The carrier gas was He and the injector and detector temperatures were both 280°C. The oven temperature was raised from 50 to 180°C at the rate of 10°C/min, and then raised to 240°C at a rate of 0.7°C/min. Individual FA methyl esters were identified by reference to well-characterized standards. The relative amount of each FA was expressed as a percentage of the total amount of FA in the analyzed sample, and the absolute amount of FA per gram of tissue was calculated using C23:0 methyl ester as the internal standard.

For lipid class separation, a sub-sample of the chloroform–methanol phase was dried under N_2_ gas, and re-dissolved in hexane (Merck, Kenilworth, NJ, USA), before being applied to a TLC silica-gel. A mixture of petroleum ether, diethyl ether and acetic acid (113:20:1 in volume) as the mobile phase was used to separate PL from other lipid classes. The lipid classes were detected after separation by spraying the TLC plate with 2% 2-7-dichlorofluorescein, and identified with known standards under 366-nm UV light. The PL fraction was scraped from the silica plates and soaked with a polar solvent (chloroform, methanol, acetic acid and water (50:39:1:10) to elute PL from the silica gel) [45]. PL classes were separated by TLC silica-gel using a mixture of chloroform, methanol, acetic acid and water (100:75:6:2 in volume) as the mobile phase. The different PL classes were quantified as FA methyl esters using GC.

### Gene expression analysis

Methods for gonadal gene expression analysis generally followed Luckenbach et al. [46]. Gonadal RNA was isolated with TRI Reagent (Molecular Research Center, Cincinnati, USA), solubilized in a 200 μl volume of nuclease-free water to a concentration of 300 ng/μl, and DNase treated using the DNA Free kit’s ‘‘rigorous” protocol (Applied Biosystems/Ambion (ABI), Austin, TX, USA). RNA yield and quality were assessed by NanoDrop (ND-1000 Spectrophotometer; NanoDrop Technologies, Rockland, DE) and gel electrophoresis. For each sample, 1 μg of total RNA was reverse transcribed in a 10-μl reaction with the Superscript II kit (Thermo Fisher Scientific, Waltham, MA, USA). Other necessary components for reverse transcription (RT), such as random primers and RNase inhibitor, were purchased from Promega (Madison, USA). Negative control reactions were performed without the addition of the RT enzyme for a subset of the RNA samples.

Primers for quantitative reverse transcription-PCR (qPCR) designs were either obtained from previous transcriptomics work or relevant studies on lipid metabolism in salmon or designed based on cDNA sequences in GenBank (Table 2). Primers were designed with MacVector software (Accelrys, San Diego, CA, USA) and purchased from Integrated DNA Technologies (Coralville, IA, USA). Assays were run on an ABI HT7900 Fast Real-Time PCR in 384-well plates using standard cycling conditions: 50°C for 2 min, 95°C for 10 min, followed by 40 cycles of 95°C for 15 s and 60°C for 1 min. Reactions were 12.5 μl each and consisted of 1x Power SYBR Green master mix (Thermo Fisher Scientific), 150 nM of the forward and reverse primer, and 1 ng cDNA template based on the amount of total RNA loaded into the RT reactions. Standard curve samples generated from a serial dilution of cDNA (from pooled RNA from the study) were included in each plate. Standard curve samples ranged from 0.1 to 10 ng cDNA, again, based on the amount of RNA added to the RT reactions, and represented 4 dilutions over this range. Linearity of the standard curve was confirmed for the assays and results were analyzed using the relative standard curve method. Each standard curve cDNA dilution was run in triplicate. All samples from an experiment were assayed in the same plate to avoid across plate variation. A dissociation or melt curve analysis step was included in each qPCR to confirm the assay specificity. Negative controls included in each plate consisted of either no cDNA template (no template control, NTC) or RNA that was not reverse transcribed (no amplification control, NAC). These controls showed no detectable amplification over 40 cycles of PCR. Results for each target gene were normalized to the reference gene (*18s*), corrected for GSI (see below), and the mean mRNA level for immature males was set to 1.0.

**Table 2:** Quantitative PCR primer sequences for targeted genes: PCR product sizes, average PCR efficiencies, and the mean Cycle Threshold (CT) values of gonadal cDNA samples from Atlantic salmon (n = 24).

Targeted gene	GenBank accession #	Forward Primer	Revers Primer	Product size (bp)	PCR efficiency (%)	Mean C_T_
*star*^*1*^	XM_014171084	GATGGGAGATTGGAACCCTAAC	ACAGCGAACACTAACGAAGTCC	139	95.6	28.29
*hsd3b*^*1*^	XM_014174054	GAGGGGGACATTAGTGATAGTGAG	GCTGGGTTCCTTTGACGTTG	142	112.8	29.75
*hsd11b*^*1*^	XM_014176669	CCCTCGTCTCACCCTACTACA	TTATTCACCAAGCCCCACAGA	114	95.6	27.71
*cyp11a (p450scc)* ^*1*^	DQ361039	CTCAAGAATGGGGAGGACTG	ACTTCATCCAACAGAGGAACAAAG	101	92.8	28.21
*cyp11b (p450c11)* ^*1*^	XM_014149090	AAAGAGGGAGGAGAGGAGGAGAG	GGAGGGAGAGGTGGAGAAGAG	135	109.0	29.27
*cyp17a (p450c17)* ^*1*^	XM_014154002	GGCTACAGGTCTTCCCCAAC	AACTTGTGTTCCTCGTATTTCTTC	97	103.3	28.79
*amh*^*1*^	NM_001123585	CAAAAACACCAGAGACAGGACAA	TATCCGTTGAGAAAAGCACCA	111	96.2	25.81
*acsl4*^*2*^	NM001173689	AGCACCTGAACAATGACTTCT	TCCTTCTTACGGTCCACTATCT	115	94.8	27.60
*acot2*^*2*^	GEGY01113449	GGACTTTCTCCGCACCAGAA	GGGAGAGAGGCACAGGTCTA	126	86.1	26.81
*lpl*^*3*^	XM_014216226	GCCCGACCTTTGAGTTTGC	ACGTCCACAAAGAGAGCATCGT	67	107.6	30.51
*lipe (hsl)*^*2*^	NM_001173663	GCCAAGAACCCGTTTGTGTC	TGGCCCATATTCCGCAGTTT	143	110.5	26.92
*cd36*^*3*^	AY606034	TTTCCTGCTGCGCACCTT	GGTGCGGGTCATGAAGATTT	70	94.8	25.10
*fatp*^*3*^	CA373015	AGGAGAGAACGTCTCCACCA	CGCATCACAGTCAAATGTCC	157	96.6	30.80
*fabp11*^*3*^	DR695475	CCGCCGACGACAGAAAAA	TTTTGCACAAGGTTGCCATTT	61	95.9	25.90
*fads1 (Δ5 desaturase)*^*4*^	AF478472	GAGAGCTGGCACCGACAGAG	GAGCTGCATTTTTCCCATGG	51	89.1	30.77
*fads2 (Δ6 desaturase)*^*4*^	AY458652	AGAGCGTAGCTGACACAGCG	TCCTCGGTTCTCTCTGCTCC	51	85.8	29.48
*elovl2*^*4*^	TC91192	CGGGTACAAAATGTGCTGGT	TCTGTTTGCCGATAGCCATT	123	104.6	29.50
*elovl5a*^*4*^	AY170327	GATGAGCGGGTACAGGGATG	CGGCTGTCTGTGTCTCATGT	117	92.4	27.38
*elovl5b*^*4*^	DW546112	ACAAAAAGCCATGTTTATCTGAAAGA	AAGTGGGTCTCTGGGGCTGTG	141	99.0	30.44
*fasn (fas)*^*2*^	XM014179803	CACCTTCGGTCATCTTCTGTT	CAGGATGGAGGAGATTGTTGTAG	123	96.6	27.09
*chpt1*^*5*^	GBRB01045139	GTACGCCTTCCCCATCTTGG	TGAGGCCGATGTGCATACCT	149	110.4	28.80
*cept1*^*5*^	NM_001139869	GCAGGCCAGACGAACCAATA	TGAACATGCCAGCGAAACAGC	161	98.4	28.78
*apoa1*^*5*^	NM_001123663	CTGGTCCTCGCACTAACCAT	TGGACCTCTGTGCAGTCAAC	142	103.5	27.57
*apob*^*6*^	X81856	TTGCAGAGACCTTTAAGTTCATTCA	TGTGCAGTGGTTGCCTTGAC	121	103.5	28.98
*18s*^*1*^	AJ427629	CTCAACACGGGAACCCTCAC	AGACAAATCGCTCCACCAAC	118	92.4	10.57

^1^ Maugars and Schmitz [16]

^2^ New assay designed for the present study

^3^ Torstensen et al [47]

^4^ Thomassen et al [48]

^5^ Carmona-Antonanzas et al [37]

^6^ Gu et al [49].

### Data normalization and statistical analysis

To account for dynamic changes in gonad size during maturation, gene expression and lipid data were corrected for GSI. Messenger RNA levels were normalized to the total testis mRNA (i.e., total gonadal mRNA level), gonad weight, and fish body weight according to prior studies of salmonid fish by Maugars and Schmitz [18] and Kusakabe et. al. [2] and calculated as follows:

$$Gonadal\;mRNA\;levels=\frac{Target\;gene\;quantity}{18s\;quantity}\times \frac{Extracted\;RNA\;amount}{Extracted\;tissue\;weight}\times \frac{Gonad\;weight}{Body\;weight}$$

For lipid data, levels of FA and PL were normalized to the total lipid content, gonad weight and body weight, similar to prior studies [12, 50] and calculated as follows:

$$Gonadal\;content\;of\;fatty\;acids=\frac{Extracted\;lipid\;quantity\;(mg)}{Extracted\;tissue\;weight\;(g)}\times \frac{Gonad\;weight\;(g)}{Body\;weight\;(kg)}$$

Data not corrected for GSI are also provided in S1 and S2 Tables.

All data were analyzed using Microsoft Excel (Microsoft Office, Redmond, WA, USA) and Statistica 7.0 (StatSoft Inc, Tulsa, OK, USA) for Windows and log transformed prior to statistical analysis. Multiple regression analysis was conducted in order to identify correlative relationships between GSI and gonadal FA or PL content, or gene expression in maturing males. Biological and analytical data were subjected to one or two-way analysis of variance (ANOVA) between immature females (IMF), immature males (IMM), and maturing males (MM). For analysis, maturing males were divided into two groups based on GSI: MM-1 was composed of fish with GSI < 5 (1.1–4.7; mean = 3.1±0.5; n = 8) and MM-2 was composed of fish with GSI > 5 (5.1–7.8; mean = 6.3±0.4; n = 6). When significant differences (*P*<0.05) among groups were identified, multiple comparisons among means were made using the Tukey post-hoc test. All data are presented as mean±SE.

## Results

### Gonadal growth and histology

Immature female and male salmon had an average GSI of 0.11±0.01 and 0.06±0.01, respectively (Table 3). In comparison, males classified as maturing had an average GSI of 4.45±2.03, with values from 1.1 to 7.8. There was thus a 73-fold increase in total gonad mass between immature and maturing males. The average body weight of immature and maturing males was similar (~5.4 kg). However, GSI was significantly higher and HSI significantly lower for both maturing male groups, while VSI was significantly lower for only the MM-2 group relative to immature males, indicating a shift in the body composition of maturing males. Among the maturing males there was no correlation between body weight and GSI.

**Table 3:** Biometric data of immature female (n = 4), immature male (n = 6) and maturing male (MM-1, n = 8; MM-2, n = 6) Atlantic salmon.

Biometric data	IMF	IMM	MM-1	MM-2	ANOVA (*P*-value)	
					M	G
Body weight (g)	5411 ± 169 ^a^	5370 ± 209 ^a^	5405 ± 334 ^a^	5590 ± 531 ^a^	0.79	0.94
VSI	6.64 ± 0.47 ^b^	6.76 ± 0.14 ^b^	5.30 ± 0.50 ^ab^	4.19 ± 0.28 ^a^	<0.01	0.11
HSI	0.81 ± 0.02 ^ab^	0.87 ± 0.02 ^b^	0.73 ± 0.02 ^a^	0.73 ± 0.03 ^a^	<0.01	0.35
GSI	0.11 ± 0.01 ^a^	0.06 ± 0.01 ^a^	3.09 ± 0.51 ^b^	6.25 ± 0.40 ^c^	<0.01	0.04
Gutted yield (%)	88.4 ± 0.5 ^b^	87.9 ± 0.2 ^b^		84.8 ± 0.2 ^a^	0.07	0.21

IMF = immature females; IMM = immature males; MM-1/-2 = maturing males (1: GSI<5 and 2: GSI>5); VSI = viceralsomatic index; HSI = Hepatosomatic index; GSI = Gonadosomatic index. Mean±SE. Statistical analysis by 2-way ANOVA, mean effect of maturation status (M; immature vs maturing) and gender (G; females vs males), followed by Tukey post hoc test were different letters denote significant differences (P<0.05) among groups.

**Fig 1:**
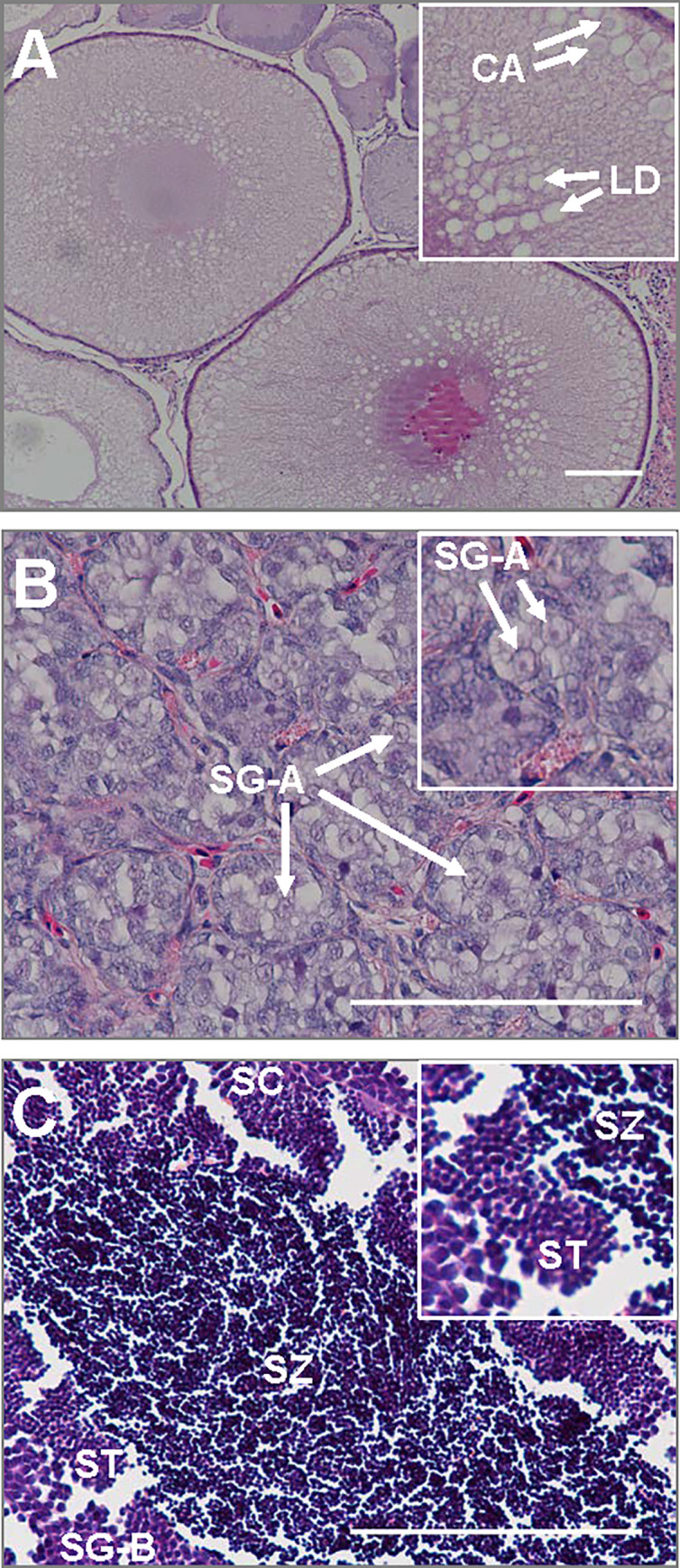
Representative histological sections of gonads sampled from Atlantic salmon (*Salmo salar*) farmed at Nordlaks AS (Norway). Shown are immature ovaries of a female (A), and immature testes (B) and sexually maturing testes of males (C) with higher magnification insets in each. Cortical alveoli (CA); early lipid droplets (LD); type-A spermatogonia (SG-A); type-B spermatogonia (SG-B); spermatocytes (SC); spermatids (ST); spermatozoa (SZ). Scale bars are 100 μm in main images; higher magnification inset widths are 150 μm for panel A and 50 μm for panels B & C.

Immature females had ovarian follicles at the late cortical-alveolus to early lipid droplet stage (Fig 1A) and likely would have initiated vitellogenesis during the following year. Immature males had testes with a homogenous distribution of type-A spermatogonia (Fig 1B). Despite having significantly different GSI values, the maturing male groups (MM-1 and -2) were in mid-spermatogenesis and had testes that exhibited of a range of germ-cell stages (i.e., spermatogonia, spermatocytes, spermatids and spermatozoa; Fig 1C). Morphology of the tubular lobes encompassing the germ cells in the testes were significantly larger in diameter in maturing males (grouped) compared to those of immature males, averaging 267±89 μm and 112±16 μm, respectively.

### Total gonadal fatty acid and phospholipid content

**Fig 2:**
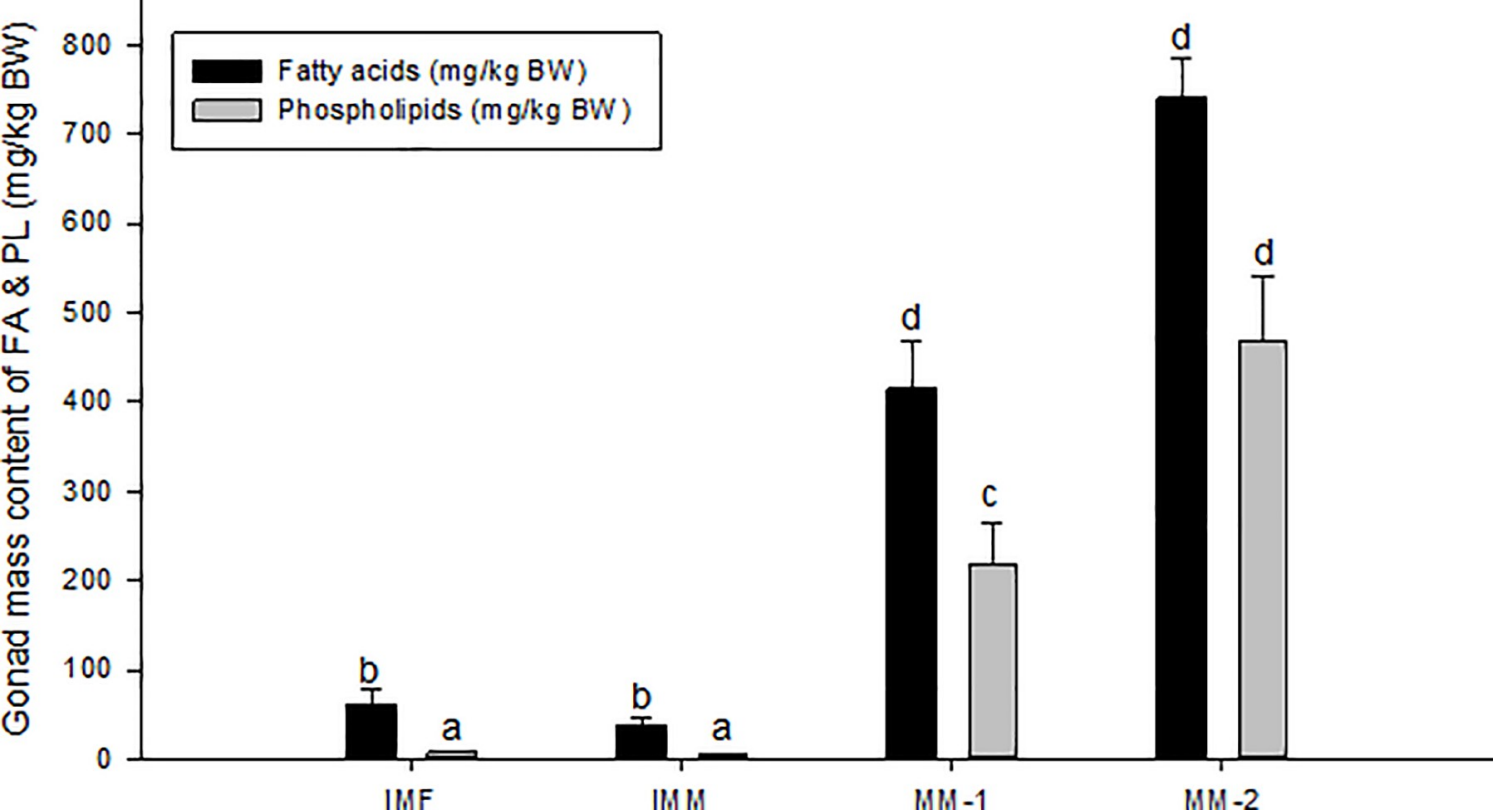
Fatty Acid (FA) and phospholipid (PL) composition in gonads of Atlantic salmon, immature females (IMF; n = 4), immature males (IMM; n = 6) and maturing male (MM-1, n = 8; MM-2, n = 6). Mean±SE. ANOVA followed by Tukey post-hoc test for pairwise comparisons. Different letters denote significant differences (P<0.05) among groups.

Both groups of maturing males had significantly higher total gonadal FA content on average than immature males and females (Fig 2). Similarly, total gonadal PL content was significantly higher in both groups of maturing males compared to immature males and females; PL content also doubled from MM-1 to MM-2. Maturing males thus had significantly (*P*<0.05) higher gonadal FA and PL content compared to immature fish. Maturing males also exhibited a reduction in the content of FA relative PL, as the ratio of total FA and total PL was significantly different between immature and maturing fish, with a ratio of ~7:1 in immature females and males, and ~2:1 for MM-1 and ~1.5:1 for MM-2 (Fig 2). This indicated a shift in overall lipid composition toward higher amounts of PL as maturation progressed.

**Fig 3:**
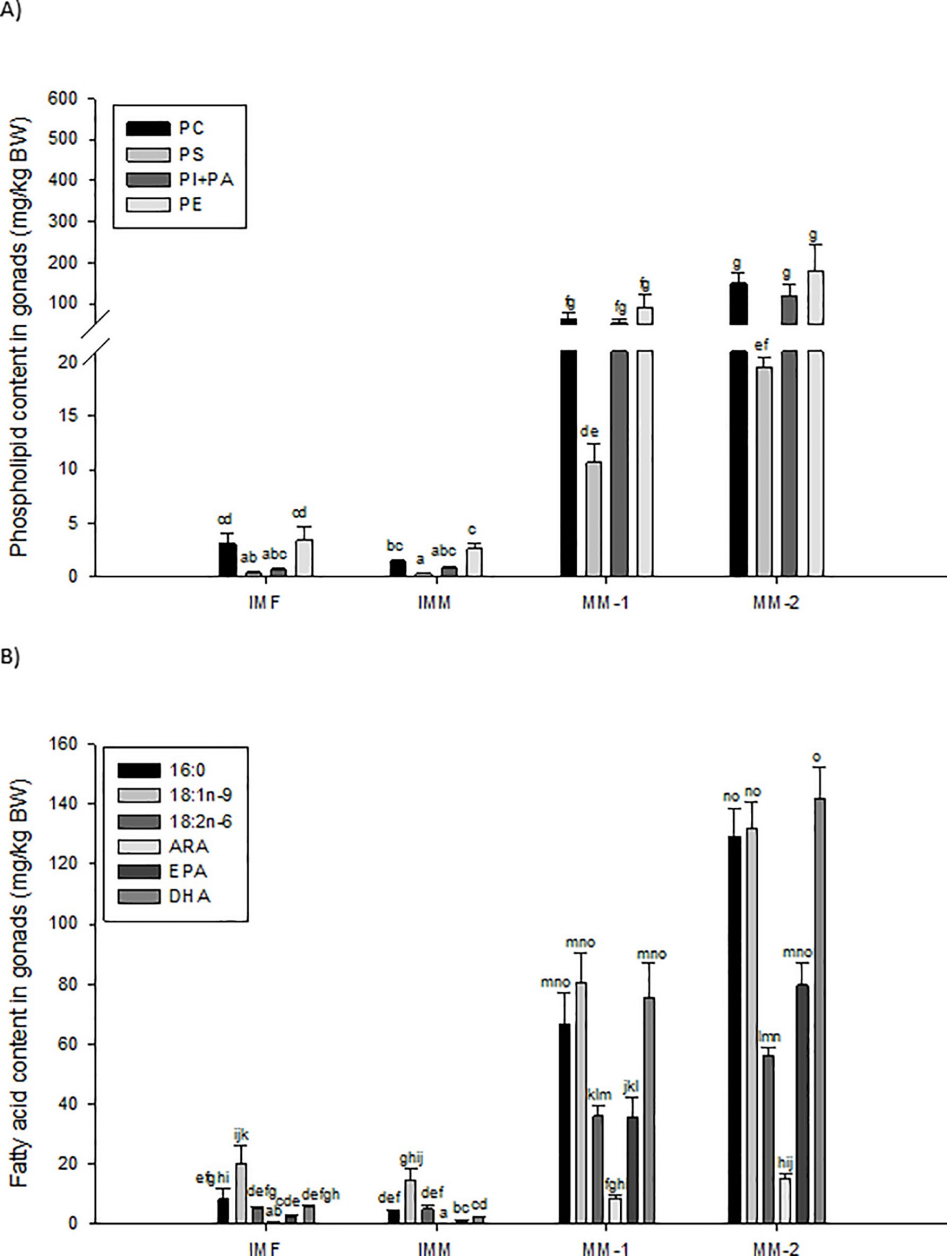
Levels of different phospholipid (PL) and Fatty Acid (FA) classes based on total gonad mass of immature females (IMF; n = 4), immature males (IMM; n = 6) and maturing male (MM-1, n = 8; MM-2, n = 6) Atlantic salmon. Mean±SE. ANOVA followed by Tukey post-hoc test was used for pairwise comparisons. Different letters denote significant differences (P<0.05) among groups. PC = Phosphatidylcholine; PS = Phosphatidylserine; PI = Phosphatidylinositol; PA = Phosphatidic acid; PE = Phosphatidylethanolamine; PL = Phospholipids; ARA = Arachidonic acid (20:4n-6); EPA = Eicosapentaenoic acid (20:5n-3); DHA = Docosahexaenoic acid (22:6n-3).

In maturing males, total gonadal content of PC, PS, PI+PA and all FA identified in the testes were significantly (*P*<0.01) and positively correlated with GSI (Table 4). The total content of FA known to be produced *de novo* (16:0) and essential LC-PUFA (e.g. ARA, EPA and DHA) showed the strongest correlations with GSI (R^2^ = 0.7–0.8). Levels of key PL and FA in the gonads of immature females and males, and maturing males are presented in Fig 3. All groups had a significantly higher total content of PC and PE compared to PS (*P*<0.01; Fig 3A). While the total content of PI+PA was not significantly different from the other PL classes in immature salmon, PI+PA was significantly higher than PS content in maturing males (*P*<0.01). Thus, relative to total content of PC and PE, there appeared to be a marked increase in testis PI+PA content during male maturation (Fig 3A, S1 Table). When considering changes in overall PL ratios of both maturing male groups there was a notable increase in PI+PA and decrease in PE (around +10% and -10%, respectively) in the gonads. The two groups of maturing males showed a slight increase, albeit not significant (*P*>0.05), in levels of all gonadal PLs with increasing GSI.

**Table 4:** Composition of Fatty Acids (FA) and phospholipids (PL) and correlation to GSI in maturing Atlantic salmon (n = 14).

FA and PL(mg/kg BW)	Mean	Min	Max	R-value^1^
PC	100	20	216	0.32*
PE	130	20	337	0.13_
PI+PA	81	20	244	0.59*
PS	14	4	23	0.60*
14:0	6	2	9	0.51*
**16:0**	93	27	170	0.74*
18:0	22	7	38	0.67*
16:1n-7	3	0	7	0.38*
18:1n-9	102	44	170	0.43*
20:1n-9	8	4	15	0.33*
18:2n-6	44	24	66	0.49*
20:3n-6	3	1	4	0.38*
**20:4n-6 (ARA)**	11	5	21	0.70*
18:3n-3	12	6	21	0.32*
**20:5n-3 (EPA)**	55	3	113	0.79*
22:5n-3	8	3	14	0.62*
**22:6n-3 (DHA)**	104	30	184	0.74*

FA = fatty acids; PL = Phospholipids; PC = Phosphatidylcholine; PS = Phosphatidylserine; PI = Phosphatidylinositol; PA = Phosphatidic acid; PE = Phosphatidylethanolamine

^1^ R-value relative to GSI

*Indicate significant correlation between FA/PL and GSI

The FA content of 16:0, 18:1n-9, 18:2n-6, EPA and DHA tended to be higher in gonads of immature females compared to those of immature males; however, no significant differences were observed (*P*>0.05, Fig 3B). Immature testes were dominated by 18:1n-9, while those of maturing males had similar levels of 16:0, 18:1n-9 and DHA. Levels of 18:1n-9 and 18:2n-6 increased 7- and 9-fold, respectively, between immature and maturing testes, while total content of 16:0, ARA, EPA and DHA increased 25-, 34-, 43- and 46-fold, respectively. Maturing males with higher GSI (MM-2) showed a specific increase in 16:0, EPA and DHA compared to 18:1:n-9 and 18:2n-6 in maturing males with a lower GSI, although the mean values of these FA between the two groups were not significant different (Fig 3B). Lipid composition was assessed as FAME in total lipid and total FAME within each PL class. Relative calculation to total FAME (mg/g total FA) resulted in values comparable to total gonadal lipid content, with increased PL and FA in maturing males compared to immature individuals (S1 Table). More specifically, content of PI+PA increased to levels similar to PC and PE, while a shift in FA composition occurred with decreased content of 16:1n-7, 18:2n-6, 18:3n-3, 20:1n-9 and increased 16:0, 18:0, ARA, EPA, 22:5n-3 and DHA (S1 Table).

### Genes associated with sexual maturation

**Fig 4:**
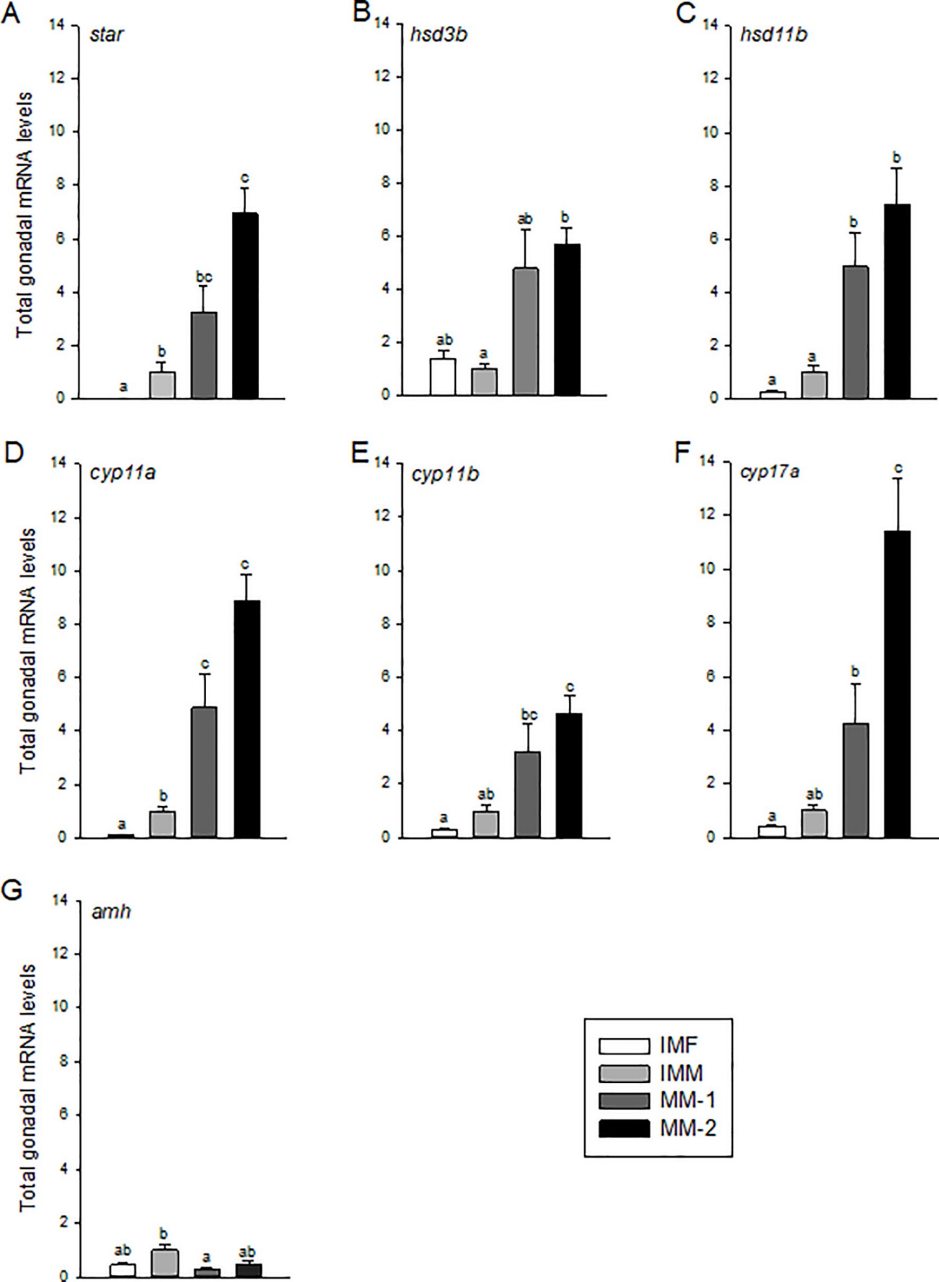
Total mRNA levels of genes associated with sexual maturation in gonads of immature females (IMF; n = 4), immature males (IMM; n = 6) and maturing male (MM-1, n = 8; MM-2, n = 6) Atlantic salmon. Different letters denote significant differences (P<0.05) among groups.

Total gonadal mRNA levels for steroidogenesis-associated genes (*star*, *hsd3b1*, *hsd11b*, *cyp11a*, *cyp11b* and *cyp17a*) were significantly elevated (5- to 10-fold) in maturing males with higher GSI (MM-2) compared to immature males or females (Fig 4A–4F), while MM-1 fish often had intermediate levels that were not always significantly elevated relative to immature fish. There were also differences between immature males and females for some genes, including *star* and *cyp11a*; however, these differences were generally minor. Total gonadal mRNA levels of *amh*, a gene that is typically down-regulated during the onset of spermatogenesis, was significantly reduced in MM-1 fish compared to immature males (Fig 4G). In maturing males, only *star* and *cyp17a* mRNA levels were significantly correlated with increasing GSI (Table 5). These genes also showed the most striking increases between MM-1 and MM-2 fish, but only data for *cyp17a* were significant (Fig 4).

**Table 5:** Testicular mRNA levels of selected genes relative to GSI in sexually maturing Atlantic salmon (n = 14).

Genes	mRNA corrected for testis mass^1^			
	Mean	Min	Max	R-values^2^
_*star*_	4.8	0.7	9.3	0.40*
_*hsd3b*_	5.2	0.4	13.5	0.04_
_*hsd11b*_	6.0	0.6	12.9	0.06_
_*cyp11a (p450scc)*_	6.6	0.8	11.8	0.28_
_*cyp11b (p450c11)*_	3.8	0.7	9.5	0.17_
_*cyp17a (p450c17)*_	7.3	0.2	19.2	0.56*
_*amh*_	0.4	0.0	0.9	0.11_
_*acsl4*_	137.1	18.2	328.0	0.09_
_*acot2*_	45.9	3.9	109.5	0.58*
_*lpl*_	779.1	57.2	1542.9	0.48*
_*lipe (hsl)*_	21.0	3.2	37.5	0.41_
_*cd36*_	21.2	4.5	43.2	0.43*
_*fatp*_	6.7	0.4	14.4	0.01_
_*fabp11*_	9.4	2.2	19.8	0.28_
_*fads1 (Δ5 desaturase)*_	11.4	2.5	19.4	0.24_
_*fads2 (Δ6 desaturase)*_	13.9	3.3	27.4	0.24_
_*elovl2*_	122.3	2.4	424.8	0.43*
_*elovl5a*_	18.6	4.5	36.3	0.14_
_*elovl5b*_	9.5	0.0	34.4	0.00_
_*fasn (fas)*_	48.3	8.6	111.9	0.15_
_*chpt1*_	7.2	1.8	16.3	0.21_
_*cept1*_	22.5	3.5	44.8	0.19_
_*apoa1*_	65.7	1.1	400.8	0.06
_*apob*_	45.2	0.9	265.5	0.04_

^1^ Values relative to mean values for immature males set to 1.0, after normalization to *18s* and correction to testis mass/body weight

^2^ R-values relative to GSI

*Indicate significant correlation between mRNA levels and GSI

### Genes involved in intracellular regulation of arachidonic acid

**Fig 5:**
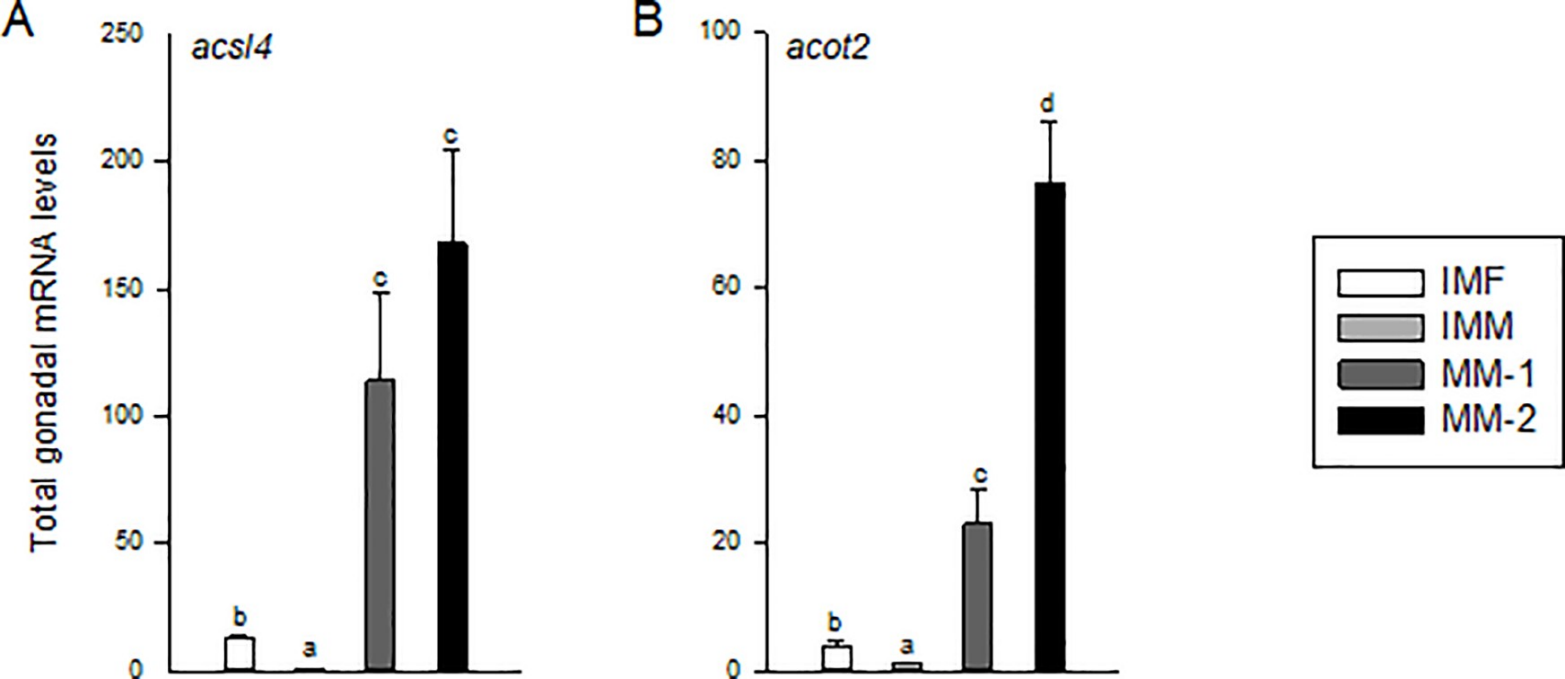
Total mRNA levels of genes involved in regulation of intracellular ARA levels in gonads of immature females (IMF; n = 4), immature male (IMM; n = 6) and maturing males (MM-1, n = 8; MM-2, n = 6) Atlantic salmon. Different letters denote significant differences (P<0.05) among groups.

The genes *acsl4* and *acot2* are involved in intercellular regulation of ARA and cholesterol import via STAR, and as noted above, both ARA and *star* levels were elevated in maturing males. Total gonadal levels of *acsl4* and *acot2* mRNA were significantly higher in maturing males compared to immature males and females (*P*<0.01; Fig 5A and 5B) with a ~150- and ~75-fold increase from IMM to MM-2 for *acsl4* and *acot2*, respectively. However, only *acot2* mRNA levels were significantly correlated with GSI in maturing males (Table 5), also reflected by significantly higher *acot2* in MM-2 compared to MM-1 fish (Fig 5B). Immature females on the other hand had significantly higher levels of *acsl4* and *acot2* compared to immature males, albeit at lower levels than those of maturing males (Figs 4 and 5).

### Gonadal lipid uptake, transport and hydrolysis

**Fig 6:**
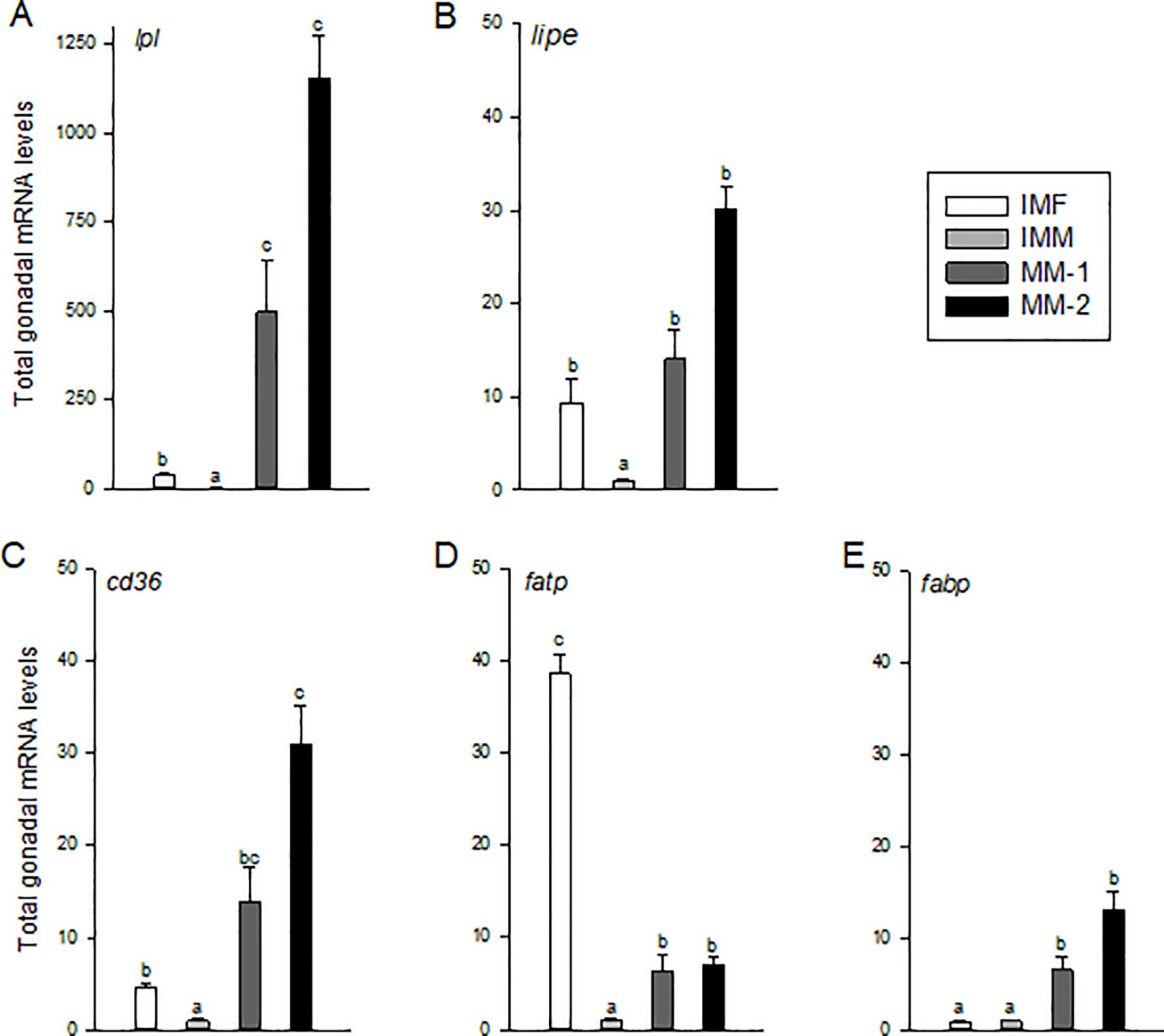
Total mRNA levels of genes associated with lipid hydrolysis and Fatty Acid (FA) uptake and transport in gonads of immature females (IMF; n = 4), immature males (IMM; n = 6) and maturing male (MM-1, n = 8; MM-2, n = 6) Atlantic salmon. Different letters denote significant differences (P<0.05) among groups.

Maturing males (MM-1 and MM-2) exhibited significantly higher total gonadal mRNA levels of *lpl*, *lipe*, *cd36*, *fatp*, and *fabp11* compared to those of immature males (Fig 6A–6E). There was an increase of ~10-30-fold in levels of *lipe*, *cd36*, *fatp* and *fabp11*, and a striking 500-1000-fold increase in levels of *lpl* in the maturing male groups compared to immature males. In addition, total gonadal levels of *cd36* and *lpl* in maturing males were significantly correlated with increasing GSI (Table 5); however, there were no significant differences between the MM-1 and -2 groups when directly compared, though levels tended to be higher in MM-2 fish (Fig 6). Total gonadal mRNA levels of *lpl*, *lipe*, *cd36* and *fatp* in immature females were significantly higher than those of immature males (Fig 6). In the case of gonadal *fatp*, levels were significantly higher in immature females than those of immature or maturing males. Levels of *fabp11* mRNA on the other hand were not significantly different among immature males or females.

### Elongation and desaturation of gonadal fatty acids

**Fig 7:**
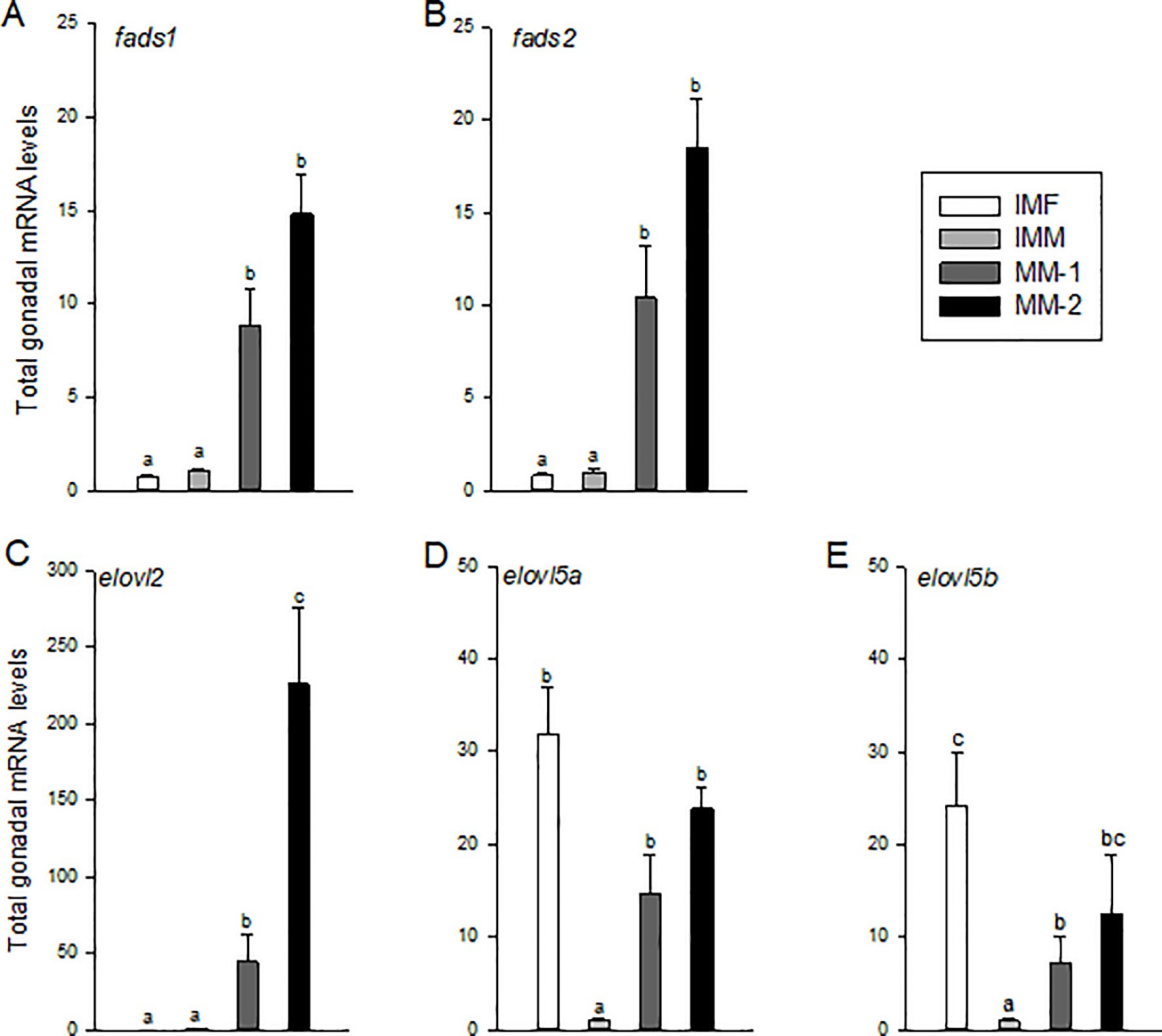
Total mRNA levels of genes associated with elongation and desaturation of Fatty Acids (FA) in gonads of immature females (IMF; n = 4), immature males (IMM; n = 6) and maturing male (MM-1, n = 8; MM-2, n = 6) Atlantic salmon. Different letters denote significant differences (P<0.05) among groups.

Total gonadal mRNA levels of genes involved in FA desaturation (*fads1 and fads2*) and elongation (*elovl2*, *elovl5a*, and *elovl5b*) were significantly elevated in maturing males compared to immature males (Fig 7A–7E). Levels were ~10-20-fold higher for these genes, except *elovl2*, which was elevated ~50-200-fold, along with being the only one of these transcripts significantly and positively correlated with GSI in maturing males (Table 5). Levels of *elolv2* were also significantly elevated in MM-2 compared to MM-1 fish (Fig 7C). Interestingly, gonadal levels of *elovl5a* and *elovl5b* in immature females were significantly higher than those of immature males and similar to or higher than those of the maturing males groups.

### Fatty acid and phospholipid synthesis, and genes encoding apolipoproteins

**Fig 8:**
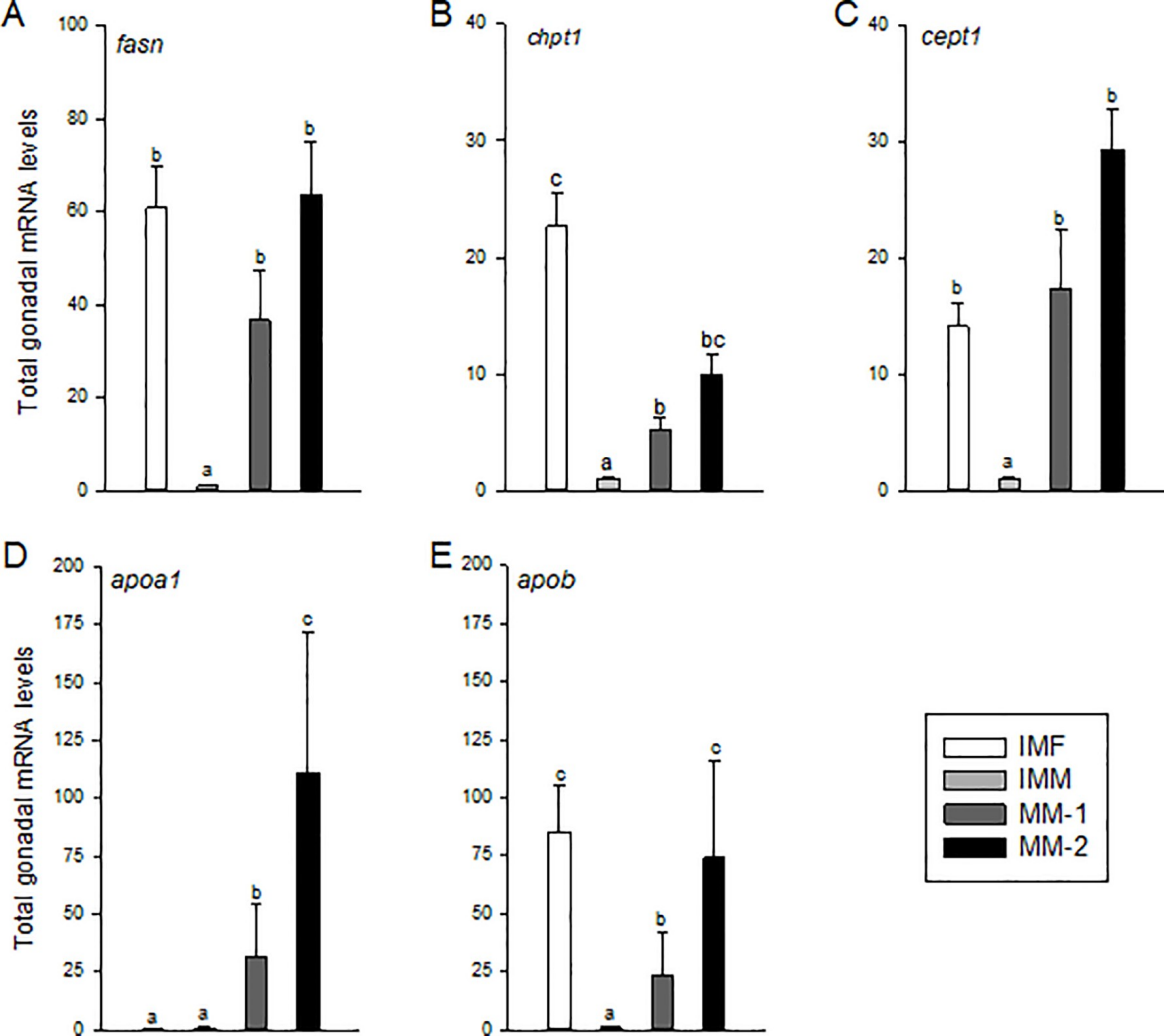
Total mRNA levels of genes associated with gonadal Fatty Acid (FA) and phospholipid (PL) synthesis, and transport of lipids from gonads of immature females (IMF; n = 4), immature males (IMM; n = 6) and maturing male (MM-1, n = 8; MM-2, n = 6) Atlantic salmon. Different letters denote significant differences (P<0.05) among groups.

Total gonadal mRNA levels of *fasn*, *chpt1* and *cept1* were significantly higher in maturing compared to immature males, with differences of ~50-fold for *fasn*, less than 10-fold for *chpt1*, and greater than 20-fold for *cept1* (Fig 8A–8C). Levels of these mRNAs, however, did not correlate with increasing GSI in maturing males (Table 5). This was also reflected by a lack of significant differences for these genes between MM-1 and MM-2 fish, though levels tended to be higher in MM-2 (Fig 8). Apolipoprotein (*apoa1* and *apob*) mRNA levels were also significantly higher in maturing compared to immature males (Fig 8). Levels of *apoa1* and *apob* mRNA were higher in MM-2 compared to MM-1 fish, although correlations for these genes with GSI were not significant (Table 5). Among the immature fish, *apoa1* was expressed at similar levels, but *fasn*, *chpt1*, *cept1*, and *apob* were significantly elevated in females and similar to maturing males (Fig 8A–8E).

## Discussion

This study investigated the testis content and composition of FA and PL and expression of key genes associated with lipid metabolism and steroidogenesis during sexual maturation in Atlantic salmon. We found that total gonadal FA and PL content was dramatically elevated in maturing testes relative to immature testes or ovaries. Increases in 16:0 and LC-PUFA levels and the PL:FA ratio, together with increased content of PI+PA (structural precursors for PC and PE), demonstrated the structural need for PL during testicular maturation. Elevated testis lipid levels were concomitant with increased expression of genes involved in FA uptake and synthesis, and production of LC-PUFA and PL. Furthermore, testis ARA levels were elevated concomitant with increased expression of genes involved in mitochondrial transport of LC-PUFA and cholesterol, which likely impact steroidogenesis, and thus sexual maturation.

In fish, analyzing changes in gonadal lipid and mRNA levels between immature and maturing testes, which undergo enormous increases size and mass during reproductive development, presents challenges that must be addressed when evaluating results. Reporting data based on a per g basis becomes less meaningful when the entire organ, and as a result, its total capacity is changing drastically, as with the ~75-fold increase in gonad size in maturing Atlantic salmon from the present study. Therefore, gonadal lipid and mRNA data were normalized for total gonadal capacity as in previous studies in fish [2, 15, 18].

Sexual maturation in fish is regulated similar to that of mammals through the pituitary release of Fsh and Lh, which bind receptors expressed in somatic cells of the gonads [51]. These gonadotropins stimulate the gonadal production of sex steroids, including estradiol-17β, testosterone and 11-ketotestosterone, which stimulate gonadal growth and development and influence the timing of maturation [reviewed by 52–55]. In male salmonids, elevated levels of plasma Fsh have been correlated with increasing 11-ketotestosterone production and GSI during gonadal development [56, 57]. Atlantic salmon naturally spawn in November and display increased levels of 11-ketotestosterone and other steroids in August (the time fish were sampled in the present study), peaking through October [58]. In steroidogenic tissues like the gonads, the rate of steroid production is attributed to the rate of cholesterol transport to the inner mitochondrial membrane, for which STAR, ARA, and the enzymes, ACSL4 and ACOT2, play critical roles [28]. The two-step sequestration of ARA to an intracellular pool by ACSL4 and intracellular transport to STAR by ACOT2 are believed to be essential to the function and regulation of STAR for transport of cholesterol into the steroidogenic pathway [26–28]. In the present study, a 30-50-fold increase in testis ARA content in maturing males was concomitant with significant increases in gonadal *acsl4* (>100-fold), *acot2* (>20-fold) and *star* mRNA (~5-fold) relative to immature males. This study also showed a particularly strong connection between testis *acot2* and *star* gene expression in males, which both exhibited significant positive correlations with increasing GSI during maturation. This was likely related to the observed significant increase in gonadal expression of steroidogenic genes and well documented increase in steroid production during male maturation of Atlantic salmon [18, 58]. Together, these data suggest that the sequestration of ARA for Star-dependent cholesterol transport is important for steroidogenesis in maturing male Atlantic salmon.

In contrast to the stimulatory effects of FSH on reproductive development, FSH is also known to suppress *amh*, a gene involved in inhibition of spermatogonial proliferation and whose decline is associated with initiation of the mitotic phase of spermatogenesis [59, 60]. Hence, testis *amh* mRNA levels have been used previously as a marker of male maturation in salmonids [61]. Consistent with prior studies, we found that testis *amh* mRNA levels were reduced with the transition from the IMM to MM-1 stage in Atlantic salmon.

We observed that the tubular compartments in maturing testes significantly increased in size as males transitioned into spermatogenesis and the testes grew in mass. Lipids are important structural and energetic constituents to support such gonadal growth. Lipids transported by plasma very low density lipoproteins (VLDL) are hydrolyzed by Lpl, releasing dietary FA that are taken up by nearby tissues as an immediate energy source or to be re-esterified for storage [13]. In comparison, LIPE (HSL) hydrolyzes intracellular neutral lipid [31] and is an important regulator of TAG reduction for increased membrane PL production to support gonadal growth during maturation. As expected, in maturing males, gonadal *lpl* and *lipe* mRNA levels were elevated relative to those of immature males. This difference was most striking for *lpl*, for which we saw the most dynamic increase of any gene measured in the study, along with significant correlation to increasing GSI and FA content. This suggests that *lpl* likely plays an important role in supplying FA to the developing Atlantic salmon testes through uptake of circulating lipids obtained from the diet and/or other tissues. This supposition agrees with previous work in female European seabass in which ovarian expression and activity of Lpl were elevated during maturation [14]. Experiments in shortfinned eel also demonstrated that treatment with the androgen 11-ketotestosterone increased ovarian *lpl* mRNA levels and lipid accumulation [15]. Thus, increases in androgen production (specifically 11-ketotestosterone) documented in Atlantic salmon [18, 58] are likely associated with the dramatic increases in *lpl* mRNA seen here, and play an important role in accumulation of testicular lipids. In comparison, the lower gonadal expression of *lipe* in the present study suggests a lesser extent of intracellular lipid hydrolysis at the relatively early stage of maturation the fish were sampled. LIPE is important, however, especially during the final stage of spermatogenesis when spermatids are elongated and the cytoplasmic reduction induces the release of membrane-bound vesicles rich in TAG [62]. Thus, further increases in *lipe* in maturing males would be expected during final maturation.

Increased absorption/hydrolysis of FA in testes of maturing males was supported by the observed increase in expression of genes (*cd36*, *fatp* and *fabp11*) responsible for FA uptake and transport. Levels of *fatp* mRNA however were still strikingly higher in gonads of immature females compared to those of maturing males. FATP is a membrane-bound transport protein that would suggest even higher uptake of FA into ovaries of immature females. This could be explained by the fact that female fish typically exhibit greater gonadal growth relative to their body weight prior to sexual maturation, and may therefore have a higher demand for lipid turnover. This idea is further supported by similar expression of genes for FA elongation and lipid transport (e.g., *elovl5* and *apob* mRNAs) between gonads of immature females and maturing males. Differences in apolipoprotein mRNA levels among these groups suggests preferential transport of specific lipids, with Apob being the major protein in VLDL for transport of lipids from the liver *to* peripheral tissues, and Apoa being the major protein in high-density lipoproteins (HDL) for transport *from* peripheral tissues. Plasma lipoprotein analysis in Atlantic salmon has previously shown a TAG:cholesterol ratio of 4:1 in VLDL and 1:1 in HDL, respectively [63]. The higher levels of gonadal *apoa1* mRNA in maturing males relative to other groups, and similar levels of *apob* mRNA between immature females and MM-2 fish in the present study may indicate a shift in preferential lipid transport during maturation. Thus, upregulation of gonadal *apob* levels suggests similar lipid transport of dietary TAG in immature females and higher-GSI maturing males, while the upregulation of *apoa1* in maturing males suggests an increase in lipid transport from peripheral tissues, likely related to heightened cholesterol demand for steroidogenesis.

Absorption of FA supports increased demand for membrane structures as the somatic and interstitial cells of the gonads proliferate during maturation [64]. In comparison to immature males, maturing males require more specific FA that can contribute to these structures. These FA could either be provided through the diet, modified through elongation and/or desaturation of FA that have been absorbed, or produced *de novo* by the gonads. Endogenous *de novo* FA production by cells is generally considered low [5–6] and thought to mainly occur in the liver [13]. In the present study, gonadal *fasn* expression and the FA, 16:0, were significantly higher in maturing relative to immature males, indicating that the testes have some capacity for *de novo* FA synthesis and that this mechanism may be contributing to the overall accumulation of testicular FA during maturation. Previous studies have demonstrated increased production of 16:0 under the influence of androgen production [21], which supports our findings. Testes of maturing males also exhibited elevated expression of genes involved in desaturation (*fads1* and *fads2*) and elongation (*elovl2*), which encode key enzymes for the production of DHA [65]. In comparison, gonadal *elovl5* mRNA levels were similar in immature females and maturing males, suggesting more efficient elongation of C18 to C20 FA compared to immature males. While 18:1n-9 was the major FA in the gonads of immature salmon, we found similar levels of 16:0, 18:1n-9 and DHA in testes of maturing males. Although this study was not designed to determine the relative contributions of *de novo* synthesis and circulating lipids to testicular development, this is an important question that should be addressed in future research. Based on our current evidence, we propose that increased endogenous production of 16:0 and elongation/desaturation to DHA, together with uptake of extra-gonadal and dietary-supplied circulating lipids via Lpl, provide the basis for increased FA content for growth and structural purposes during testicular maturation in salmon.

Membrane synthesis is required for the production of spermatozoa and to maintain sperm quality [36, 37]. Phospholipids are the main structural components of these membranes in mammals, as well as in fish [41], and the present study showed that PL are increasingly produced during sexual maturation. Messenger RNA levels of genes involved in the production of PC and PE (*chpt1* and *cept1*, respectively) were significantly higher in gonads of maturing compared to immature males, but not compared to immature females. The increase in testis content of PI+PA in maturing males supports an increasing need for these membrane structures during maturation. The major FA in PC are 16:0 and DHA [12, 13], which we found are increasingly produced during male maturation. The regulation of FA biosynthesis and exchange of FA among intracellular organelles of germ cells during spermatogenesis are essential for male fertility [62]. Lipid peroxidation activity of unsaturated FA on the sperm cell membrane have been suggested to promote binding to the egg during fertilization [66]. Therefore, increased PL and LC-PUFA content in testes of maturing males may be related to both structural support of cell membranes and enhancement of sperm quality.

In summary, this study showed elevated levels of ARA in Atlantic salmon testes during sexual maturation, which likely stimulates sex steroid production via increased gonadal *star* gene expression and cholesterol transport into the mitochondrial steroidogenesis pathway. Increased testicular *lpl* and *lipe* mRNA levels were observed concomitant with increases in FA content. Further elongation and/or desaturation to DHA and *de novo* synthesis of 16:0 supports targeted production of PL, likely related to structural growth and development of the testes during maturation. However, the significance of endogenous production of these FA must be studied further. Our findings suggest similar lipid production and function in Atlantic salmon as previously observed during testicular maturation in mammals. This study sets the stage for further experiments targeting the precise physiological mechanisms at play during testicular growth and development and the importance of dietary lipids for stimulation or inhibition of sexual maturation in Atlantic salmon aquaculture.

## Supporting information

S1 Table Fatty Acids (FA) and phospholipids (PL) in gonads of Atlantic salmon. (PDF)

S2 Table Gonadal mRNA levels of selected genes in Atlantic salmon. (PDF)
