# Supplementary material for: Phospholipid and LC-PUFA metabolism in Atlantic salmon (Salmo salar) testes during sexual maturation
Source: PLoS One. 2020 May 29;15(5):e0233322. doi: 10.1371/journal.pone.0233322 (PMC7259613; doi:10.1371/journal.pone.0233322)
Supplement: S1 Table — (PDF) [file pone.0233322.s001.pdf]

S1 Table. Fatty acids (FA) and phospholipids (PL) in gonads of immature females (IMF; n=4), immature males (IMM; n=6) and maturing males (MM-1, n=8; MM-2, n=6) Atlantic salmon.

FA and PL	(mg/g gonad)				(mg/kg BW)				(mg/g total FA)			
	IMF	IMM	MM-1	MM-2	IMF	IMM	MM-1	MM-2	IMF	IMM	MM-1	MM-2
PC	3.17^{a}	2.46^{a}	2.03^{a}	2.46^{a}	3.1^{a}	1.5^{a}	61.7^{b}	150.6^{c}	7.60^{ab}	4.53^{a}	14.28^{bc}	20.31^{c}
PE	3.50^{a}	4.63^{a}	2.94^{a}	3.01^{a}	3.4^{a}	2.6^{a}	91.8^{b}	180.8^{b}	8.02^{a}	8.54^{a}	20.28^{a}	24.38^{a}
**PI+PA**	**0.83^{a}**	**1.27^{ab}**	**1.76^{b}**	**1.81^{ab}**	**0.6^{a}**	**0.8^{a}**	**53.5^{b}**	**118.3^{b}**	**1.57^{a}**	**2.27^{a}**	**12.86^{b}**	**15.71^{b}**
PS	0.39^{ab}	0.52^{b}	0.34^{a}	0.31^{a}	0.4^{a}	0.3^{a}	10.6^{b}	19.5^{c}	0.76^{a}	1.01^{a}	2.47^{b}	2.65^{b}
Sum PL	7.40^{a}	8.89^{a}	7.07^{a}	7.60^{a}	7.5^{a}	5.2^{a}	217.7^{b}	469.2^{c}	16.06^{a}	16.36^{a}	49.90^{b}	63.05^{b}
14:0	1.45^{b}	1.09^{b}	0.16^{a}	0.12^{a}	1.8^{a}	0.7^{a}	4.7^{b}	7.7^{b}	2.28^{b}	1.78^{b}	1.15^{a}	1.04^{a}
**16:0**	**7.17^{b}**	**6.12^{b}**	**2.18^{a}**	**2.06^{a}**	**8.3^{a}**	**3.9^{a}**	**66.7^{b}**	**129.2^{c}**	**12.44^{a}**	**10.42^{a}**	**15.69^{b}**	**17.42^{b}**
18:0	1.52^{b}	1.90^{b}	0.53^{a}	0.49^{a}	1.7^{a}	1.2^{a}	16.4^{b}	30.4^{c}	2.67^{a}	3.28^{ab}	3.86^{bc}	4.11^{c}
16:1n-7	1.41^{b}	1.07^{b}	0.11^{a}	0.09^{a}	1.7^{a}	0.7^{ab}	3.1^{bc}	5.2^{c}	2.32^{b}	1.75^{b}	0.79^{a}	0.70^{a}
18:1n-9	18.21^{b}	22.67^{b}	2.77^{a}	2.15^{a}	20.3^{a}	14.7^{a}	80.3^{b}	131.9^{b}	33.06^{b}	37.07^{b}	19.51^{a}	17.90^{a}
20:1n-9	1.75^{b}	2.19^{b}	0.23^{a}	0.17^{a}	2.0^{a}	1.4^{a}	6.8^{b}	10.6^{b}	3.04^{b}	3.56^{b}	1.64^{a}	1.44^{a}
18:2n-6	4.89^{b}	7.67^{b}	1.31^{a}	0.91^{a}	5.1^{a}	5.0^{a}	35.9^{b}	55.8^{b}	9.66^{ab}	12.50^{b}	9.03^{a}	7.58^{a}
20:3n-6	0.14^{b}	0.20^{b}	0.08^{a}	0.05^{a}	0.1^{a}	0.1^{a}	2.2^{b}	3.2^{b}	0.41^{ab}	0.35^{a}	0.55^{b}	0.44^{ab}
**20:4n-6 (ARA)**	**0.55^{b}**	**0.55^{b}**	**0.31^{a}**	**0.24^{a}**	**0.6^{b}**	**0.3^{a}**	**8.7^{c}**	**15.2^{d}**	**1.10^{a}**	**1.03^{a}**	**2.12^{b}**	**2.04^{b}**
18:3n-3	1.82^{b}	2.77^{b}	0.34^{a}	0.25^{a}	1.9^{a}	1.8^{a}	9.8^{b}	15.3^{b}	3.61^{bc}	4.47^{c}	2.43^{ab}	2.08^{a}
**20:5n-3 (EPA)**	**2.45^{c}**	**2.06^{bc}**	**1.09^{a}**	**1.27^{ab}**	**2.6^{a}**	**1.3^{a}**	**35.6^{b}**	**79.8^{c}**	**4.91^{ab}**	**3.65^{a}**	**8.04^{bc}**	**10.72^{c}**
22:5n-3	0.65^{b}	0.66^{b}	0.22^{a}	0.18^{a}	0.7^{b}	0.4^{a}	6.2^{c}	11.3^{d}	1.29^{ab}	1.13^{a}	1.52^{b}	1.53^{b}
**22:6n-3 (DHA)**	**5.43^{c}**	**3.65^{b}**	**2.45^{a}**	**2.26^{a}**	**5.7^{b}**	**2.3^{a}**	**75.3^{c}**	**141.7^{d}**	**10.92^{b}**	**6.51^{a}**	**17.61^{c}**	**19.14^{c}**
Sum SFA	10.74^{b}	9.60^{b}	2.99^{a}	2.75^{a}	12.5^{a}	6.1^{a}	91.3^{b}	171.8^{c}	18.38^{ab}	16.30^{a}	21.53^{bc}	23.18^{c}
Sum MUFA	26.08^{b}	31.01^{b}	3.19^{a}	4.15^{a}	29.4^{a}	20.1^{a}	118.5^{b}	196.0^{b}	46.64^{b}	50.73^{b}	29.06^{a}	26.56^{a}
Sum n-6 PUFA	5.95^{b}	9.05^{b}	1.94^{a}	1.38^{a}	6.2^{a}	5.8^{a}	53.3^{b}	84.8^{b}	11.79^{a}	14.94^{a}	13.35^{a}	11.50^{a}
Sum n-3 PUFA	10.85^{b}	9.77^{b}	4.27^{a}	4.05^{a}	11.4^{a}	6.2^{a}	131.5^{b}	253.5^{c}	21.68^{a}	16.88^{a}	30.78^{b}	34.19^{b}
Sum FA	54.55^{b}	60.10^{b}	14.21^{a}	11.92^{a}	60.7^{a}	38.6^{a}	414.3^{b}	739.9^{c}	98.49^{ab}	98.86^{b}	94.71^{a}	95.43^{ab}

IMF=immature females; IMM=immature males; MM-1/-2= maturing males (1. GSI<5 and 2. GSI>5). Mean-values. Statistical analysis by 1-way ANOVA, followed by Tukey post hoc test were different letters denote significant differences (P<0.05) among groups.
