# Supplementary material for: Phospholipid and LC-PUFA metabolism in Atlantic salmon (Salmo salar) testes during sexual maturation
Source: PLoS One. 2020 May 29;15(5):e0233322. doi: 10.1371/journal.pone.0233322 (PMC7259613; doi:10.1371/journal.pone.0233322)
Supplement: S2 Table — (PDF) [file pone.0233322.s002.pdf]

Table S2. Gonadal mRNA levels of selected genes in immature females (IMF; n=4), immature males (IMM; n=6) and maturing males (MM-1, n=8; MM-2, n=6) Atlantic salmon.

Genes	mRNA uncorrected [1b]				mRNA corrected [1c]			
	IMF	IMM	MM-1	MM-2	IMF	IMM	MM-1	MM-2
star	0.01^{a}	1.00^{c}	0.13^{b}	0.13^{bc}	0.01^{a}	1.00^{b}	3.24^{bc}	6.97^{c}
hsd3b	1.03^{b}	1.00^{b}	0.19^{a}	0.11^{a}	1.39^{ab}	1.00^{a}	4.80^{ab}	5.70^{b}
hsd11b	0.20^{a}	1.00^{b}	0.21^{a}	0.15^{a}	0.27^{a}	1.00^{a}	4.96^{b}	7.31^{b}
cyp11a (p450scc)	0.09^{a}	1.00^{b}	0.19^{a}	0.16^{a}	0.12^{a}	1.00^{b}	4.88^{c}	8.89^{c}
cyp11b (p450c11)	0.23^{b}	1.00^{c}	0.12^{a}	0.08^{a}	0.31^{a}	1.00^{ab}	3.23^{bc}	4.65^{c}
cyp17a (p450c17)	0.32^{ab}	1.00^{b}	0.15^{a}	0.18^{a}	0.41^{a}	1.00^{ab}	4.23^{b}	11.40^{c}
amh	0.33^{b}	1.00^{b}	0.01^{a}	0.01^{a}	0.45^{ab}	1.00^{b}	0.28^{a}	0.49^{ab}
acsl4	8.90^{c}	1.00^{a}	4.01^{b}	3.46^{b}	12.38^{b}	1.00^{a}	113.83^{c}	168.12^{c}
acot2	**2.92^{b}**	**1.00^{a}**	**0.98^{a}**	**1.42^{ab}**	**3.94^{b}**	**1.00^{a}**	**22.97^{c}**	**76.49^{d}**
lpl	**26.33^{b}**	**1.00^{a}**	**17.07^{b}**	**21.89^{b}**	**35.71^{b}**	**1.00^{a}**	**496.56^{c}**	**1155.72^{c}**
lipe (hsl)	7.26^{b}	1.00^{a}	0.59^{a}	0.61^{a}	9.38^{b}	1.00^{a}	14.03^{b}	30.25^{b}
cd36	3.56^{c}	1.00^{b}	0.52^{a}	0.55^{ab}	4.68^{b}	1.00^{a}	13.89^{bc}	30.97^{c}
fatp	29.78^{c}	1.00^{b}	0.26^{a}	0.14^{a}	38.53^{c}	1.00^{a}	6.40^{b}	7.08^{b}
fabp11	0.65^{b}	1.00^{b}	0.26^{a}	0.30^{a}	0.88^{a}	1.00^{a}	6.53^{b}	13.13^{b}
fads1 (Δ5 desaturase)	0.52^{ab}	1.00^{b}	0.36^{a}	0.26^{a}	0.71^{a}	1.00^{a}	8.80^{b}	14.84^{b}
fads2 (Δ6 desaturase)	0.57^{ab}	1.00^{b}	0.39^{a}	0.33^{a}	0.79^{a}	1.00^{a}	10.41^{b}	18.52^{b}
elovl2	**0.20^{a}**	**1.00^{b}**	**1.67^{b}**	**4.07^{b}**	**0.25^{a}**	**1.00^{a}**	**44.51^{b}**	**225.91^{c}**
elovl5a	24.24^{c}	1.00^{b}	0.56^{a}	0.54^{ab}	31.84^{b}	1.00^{a}	14.71^{b}	23.85^{b}
elovl5b	18.11^{c}	1.00^{b}	0.29^{a}	0.44^{ab}	24.22^{c}	1.00^{a}	7.26^{b}	12.55^{bc}
fasn (fas)	45.00^{b}	1.00^{a}	1.31^{a}	1.35^{a}	60.96^{b}	1.00^{a}	36.70^{b}	63.83^{b}
chpt1	17.04^{c}	1.00^{b}	0.22^{a}	0.22^{a}	22.66^{c}	1.00^{a}	5.18^{b}	9.90^{bc}
cept1	10.89^{b}	1.00^{a}	0.65^{a}	0.59^{a}	14.08^{b}	1.00^{a}	17.37^{b}	29.35^{b}
apoa1	0.24^{a}	1.00^{a}	0.98^{a}	3.14^{a}	0.37^{a}	1.00^{a}	31.85^{b}	110.88^{c}
apob	57.14^{b}	1.00^{a}	0.72^{a}	2.28^{a}	85.24^{c}	1.00^{a}	23.38^{b}	74.24^{c}

[1] Mean qPCR quantity after normalization to *18s* (b) and correction to gonad mass/body weight (c), mean values for immature males set to 1.0 for both calculations. IMF=immature females; IMM=immature males; MM-1/-2= maturing males (1. GSI<5 and 2. GSI>5). Statistical analysis by 1-way ANOVA, followed by Tukey post hoc test were different letters denote significant differences (P<0.05) among groups.
